# Points from Hospital Reports

**Published:** 1923-08

**Authors:** 


### POINTS FROM HOSPITAL REPORTS.

### Guy's Hospital.

The work of Guy's was maintained in full activity during 1922, but in the words of the treasurer, " no outstanding event marks the history of the year." Turning to finance we find, mainly because of the abnormally small yield of legacies—that notoriously capricious source of income—that, but for King Edward's Fund's contribution of £14,070 on account of the Hospitals of London combined appeal, there would have been a deficiency of some £7,000. It is to be hoped that next year, when this source will not be available, the tide of legacies will be at full flood and that other sources will contribute abundantly. The continued reduction in the cost of living is reflected in the expenditure, which was lower by some 16 per cent. than in 1921. The report contains a noteworthy "Appeal for the Endowment of Medical Education and Research" with the object of providing a permanent income for the Medical School, the sum derived from students' fees being inadequate for its efficient maintenance. While affirming that "great as are the benefits bestowed on the individual patients, it can hardly be doubted that greater benefits are conferred upon the community by the scientific discoveries made in the wards and freely given to the world through the medium of the Medical School," the appeal recognises that the Hospital and Medical School are separate but mutually helpful institutions. We believe this to be a wise recognition in the best interests of both hospital and school. A gold medal for sick room cookery at the Westminster Exhibition of Cookery, and challenge cups in swimming and lawn tennis, are eloquent testimonies to the qualities of present-day nurses.

### St. Mary's Hospital, Paddington.

Amongst much that is noteworthy in this report, the point of arresting—perhaps even unique—interest is the announcement of the gift to the hospital, by its late medical superintendent, Dr. Mitchell Bird, who died in August last, of practically the whole of his estate, amounting to some £9,000. Incidentally, it is not a little remarkable that a medical officer, long and intimately associated with all the activities of his *alma mater*, should thus wholeheartedly have displayed his sympathy with the charitable, rather than the educational, part of its work. Few hospitals have such a precious possession as this gift. The utmost should be made of it, and this, we venture to suggest, may best be done by investing the £9,000—despite a present debt of £20,000—to create a permanent endowment of the work which the donor loved so well and in which he laboured so long, thus perpetuating not alone the memory of the gift but the substance of it.

### Queen Charlotte's Hospital.

The financial position of Queen Charlotte's is a very anxious one, as the hospital debts in spite of grants made from the sum collected by the Hospitals of London Combined Appeal, exceeds £20,000. For the first time with one exception since 1913, however, the income exceeded the expenditure, which is a hopeful sign. The number of married women treated continues to increase, and it is interesting to note that the percentage of married women in 1922 was 78 per cent., as compared with 53 per cent. twenty years ago. The demand for private nurses has been greater than ever, which shows that the hospital well maintains its high reputation as a training school.

### Mildmay Memorial Hospital.

The Mildmay Memorial Hospital reports a quiet year from the surgical and medical point of view. The total number of patients treated was 227 and 88 operations were performed. Heavy extra expenditure was incurred in erecting balconies with proper means of escape from fire, in repainting, and in outside drainage work. The accounts, however, show only a small overdraft of £34.

### National Hospital for Diseases of the Heart.

It is a pleasant thing to find a hospital with a surplus for two years running, which is the happy case with this institution. When it is found that the cause of the surplus is likely to be lasting, the pleasure is, of course, complete. Such a state of things is, however, rare, and the authorities of the National Hospital should not repine because it is denied them in this instance. Indeed, they have other sound cause for satisfaction, for, although their solvent state is mainly due to the receipt of £4,500 (more than a third of their total income) from the Ministry of Pensions for the treatment of ex-soldiers, yet a substantial contributary cause lies in the amount of other payments from or on behalf of patients, which nearly reaches £3,000. This is not only good in itself, but should prove a powerful plea in recruiting the ranks of annual subscribers.

### The City of London Maternity Hospital.

The great importance and value of the work of the Consultative Maternity and Ante-Natal Departments, as demonstrated by the decreasing number of abnormal confinements, is specially commented on. Evidence of this kind is certainly useful and encouraging, more especially because of the confidence it is calculated to give to patients who are apt to be sceptical of the utility of precautions to which they have not been accustomed. It is to be regretted that no such definite evidence can be given in the child's welfare department, where the number of attendances decreased, compared with 1921, by 10 per cent. The hospital is to be congratulated on the success of its financial administration, as shown on the one hand by the continued and enhanced success of its Appeal Committee, under the chairmanship of Mrs. Joseph Gluckstein, and on the other by a material reduction in the ordinary expenditure.

### Manchester Royal Infirmary.

The report of the Manchester Royal Infirmary for 1922 states that its income for that year fell short of the required amount—£112,000 is the ordinary annual expenditure—by £12,465. It has therefore been necessary to realise some of the capital available for general purposes. The donations included a gift of £5,000 by Mr. Robert McDougall for the equipment of the new X-ray department, as announced in our last issue. The contributions by patients are £615 less than the preceding year, and the decrease is attributed to the prevalent and prolonged unemployment. The General Superintendent and Secretary, Mr. Frank G. Hazell, will be glad to send copies of the report to Hospital Secretaries and others interested.

### The Royal Victoria Infirmary, Newcastle-upon-Tyne.

The Royal Victoria Infirmary stands on twenty-five acres of ground, and contains 534 beds in which 11,411 patients were treated during the year. Its out-patients numbered nearly 100,000, and they made 305,000 attendances; its expenditure amounted in round figures to £93,000, which was more than covered by the income, of which nearly half was provided by workpeople's and patients' contributions. This is an institution of which any city in the world might be proud. The report is a book of 255 pages, to which the table of contents is not a perfect guide. A feature of the book is its advertisements, which we see brought in £95 last year. In 1922 the work of the year was greater than ever, while watchful administration, aided by the fall in prices, resulted in a reduction of more than £12,000 in the expenditure. Mr. R. H. P. Orde retired during the year from the office of house governor and secretary after twenty-two years' service. As a mark of the "very high esteem in which he was held," Mr. Orde was presented with a service of silver and a cheque and appointed an Honorary Life Governor.

### Royal Infirmary, Sunderland.

The annual report of the Royal Infirmary, Sunderland, shows an income of £42,711 and an expenditure of £45,593, making a deficit of £2,882. This sum would have been considerably higher but for a grant of £2,335 made to the institution this year from the Voluntary Hospitals Commission. Two new departments have been opened, an orthopædic and a pathological. To the former, where injured workmen form the majority of the patients, a special contribution is being given by the workmen themselves, in addition to their weekly subscription to the Infirmary. The pathological department has also added considerably to the general efficiency of the institution, and is of great advantage to the Public Health work of the town and district.

